# The Pin Sensation in the Throat

**Published:** 1892-10

**Authors:** 


THE PIN SENSATION IN THE THROAT —(*New York Med. Jour.*, June 11, 1892.)—Dr. John Dunn reports five cases of the above condition. In the first case reported at some length, the patient had the sensation of a pin in the throat on swallowing. The sensation was referred to the right side, on a level with the deepest part of the hyoid fossa. After treating various conditions without effect, touching a small red elevated area at the back of the pharynx was found to cause the sensation. An interesting point was that this spot was described as lower down than various points really below it. Galvano-cauterization gave relief only while the wound was healing. The second patient had a similar sensation on the on the left side of the throat caused by a small somewhat inflamed "granulation" above the enlarged left tonsil. The other three cases were all caused by small hypertrophied masses of lymphoid tissue in some part of the pharynx. The interesting point in all these cases is the fact that the patients all erred in placing the cause of the sensation lower than it really was. Treatment appears to have been successful in all but the first case.—*Ex.*

